# Assessment of miRNA 106a-5p and 375-3p Expression in the Context of the Wnt/β-Catenin Pathway—Comparison of Prostate Adenocarcinoma and Benign Prostatic Hyperplasia Tissues

**DOI:** 10.3390/ijms262412073

**Published:** 2025-12-15

**Authors:** Magdalena Smereczańska, Natalia Domian, Grzegorz Młynarczyk, Irena Kasacka

**Affiliations:** 1 Department of Histology and Cytophysiology, Medical University of Bialystok, Mickiewicza 2C Street, 15-222 Bialystok, Poland; magdalena.smereczanska@umb.edu.pl (M.S.); natalia.domian@umb.edu.pl (N.D.); 2 Department of Urology, Medical University of Bialystok, M. Skłodowskiej-Curie 24A Street, 15-276 Bialystok, Poland; grzegorz.mlynarczyk@umb.edu.pl

**Keywords:** prostate adenocarcinoma, miRNAs, β-catenin, Fzd8, Wnt5a, cyclin D1

## Abstract

Prostate adenocarcinoma is mainly diagnosed based on serum PSA levels, but elevated PSA levels can also be caused by BPH, which weakens its specificity. Recent scientific studies have demonstrated that specific microRNAs regulate cancer cell proliferation by modulating the Wnt/β-catenin pathway. To date, no published literature has provided a comprehensive assessment of the interactions between miR-106a-5p and miR-375-3p and components of the Wnt/β-catenin pathway in prostate cancer. Therefore, the aim of the present study was to perform a pilot evaluation of the expression of miRNAs 106a-5p and 375-3p, as well as β-catenin, Fzd8, Wnt5a, and cyclin D1 in prostate adenocarcinoma compared with BPH. The study material consisted of samples collected from 30 patients with prostate cancer and 30 with BPH. Protein expression was analyzed using IHC and qRT-PCR methods, while miRNA levels were quantified by dPCR. Our study results revealed lower immunoreactivity and expression of genes encoding β-catenin, Fzd8, Wnt5a, and cyclin D1 and significantly higher fluorescence intensity of miRNA 106a-5p and 375-3p with prostate adenocarcinoma compared to BPH. These parallel alterations in miRNA expression and Wnt/β-catenin-related components reflect disease-specific expression patterns and warrant further investigation in larger cohorts to determine their potential utility as diagnostic biomarkers in prostate diseases.

## 1. Introduction

Since prostate adenocarcinoma is the most common cancer occurring in male patients and the second leading cause of cancer-related mortality in developed nations, early detection of this cancer is essential for effective treatment. Despite the progress that has been made in uro-oncology, the insufficient precision of available diagnostic tools remains an obstacle to the treatment of prostate cancer [1]. Prostate adenocarcinoma diagnosis is based on a combination of clinical, biochemical and imaging data. A key screening tool remains the determination of prostate-specific antigen (PSA) levels in serum. PSA is a protein produced by prostate epithelial cells under both physiological and pathological conditions. Although elevated levels are not specific for cancer—they can also occur in benign prostatic hyperplasia (BPH), prostatitis, or following urological procedures—it remains an important element of the diagnostic algorithm. In clinical practice, in patients with an elevated PSA and abnormal digital rectal examination results, further diagnostic imaging (MRI) is considered. If a malignant lesion is suspected, a prostate biopsy is performed, either standard TRUS or guided by MRI. Biopsy material is subjected to histopathological evaluation, which remains the gold standard in prostate cancer diagnosis. The evaluation is based on the Gleason grading system (currently the ISUP system), which allows for the assessment of the tumor malignancy [1]. The greatest challenge in clinical practice is accurate differentiation of prostate adenocarcinoma from BPH, due to the differences in treatment approaches and management strategies. Studies comparing adenocarcinoma to BPH have significant translational value because they enable the translation of fundamental research results into practical diagnostic and therapeutic methods that can be directly applied. Furthermore, the use of the tissues from patients with BPH as control group in prostate cancer studies is valuable, as it reflects the everyday challenges faced by urologists.

Studies have shown that the Wnt signaling pathway is involved in the development of prostate cancer. Autocrine and paracrine signaling in cancer cells from the tumor microenvironment have been shown to be involved in tumor development and metastasis. The canonical Wnt pathway regulates the survival of prostate adenocarcinoma stem cells, while the non-canonical Wnt pathway is involved in imparting invasive characteristics to cancer cells, which may be important in the early stages of carcinogenesis. Blocking individual elements of the Wnt pathway, particularly those that inhibit β-catenin interactions with key transcriptional factors, may prove to be an important therapeutic strategy for patients with prostate cancer [2].

Binding of a specific Wnt protein to the FZD receptor activates β-catenin-dependent (canonical) or β-catenin-independent (non-canonical) signaling pathways [2]. Previous studies have demonstrated that Fzd8 plays a promotive role in tumor cell proliferation and metastasis in renal and thyroid cancers, as well as contributes to bone metastasis in prostate cancer [3,4]. Cyclin D1 is a key regulator of cell cycle progression and has been implicated in the initiation and progression of various cancers. Its increased expression is commonly associated with poor prognosis and therapeutic resistance in prostate adenocarcinoma [5].

The discovery of small, non-coding RNA molecules called microRNAs has significantly expanded our knowledge of cancer pathogenesis. Their action involves post-translational silencing of genes, including those responsible for cell differentiation, proliferation and apoptosis. Depending on the function performed by specific mRNAs, microRNA molecules can act as oncogenes, controlling the course of the cell cycle, or tumor suppressors, causing the cycle to stop. Recent studies have shown a common change in the expression of these molecules in cancer cells, which has become the basis for searching for methods to modulate activity and use them in therapy. The use of microRNAs as noninvasive biomarkers in predicting the development of cancer is also important. Two miRNAs of particular interest are miR-106a-5p and miR-375-3p [6,7,8].

Literature data indicate that miR-106a-5p expression is significantly associated with the progression of prostate adenocarcinoma and correlates with an increased risk of malignancy [7,8,9]. miR-106a-5p may act as an oncomiRNA, modulating the expression of Wnt pathway-related proteins, such as β-catenin, Fzd8 and Wnt5a, as well as influencing cell cycle regulation, including cyclin D1. While miR-106a-5p appears to promote prostate adenocarcinoma progression through increased cell proliferation, recent studies highlight the contrasting role of miR-375, which acts as a suppressor and inhibitor of proliferation. MiR-375 has been shown to inhibit metastasis and proliferation in bladder cancer cells, as demonstrated in the T24 xenograft mouse model. MiR-375-3p blocked the Wnt signaling cascade and downstream molecules such as c-Myc and cyclin D1 by inhibiting the expression of Fzd8 [3,5].

We focused on the Wnt/β-catenin signaling pathway because of its well-documented role in prostate cancer initiation, progression and maintenance of prostate cancer stem cells. Dysregulation of the Wnt signaling pathway influences cell proliferation, invasion and therapy resistance in urological malignancies, making these pathway components attractive candidates for comparative analysis of BPH and prostate adenocarcinoma. In this pathway-focused analysis, candidate miRNAs were selected through a focused literature review.

Although several studies have reported dysregulation of miR-106a-5p or miR-375-3p in prostate cancer, the available evidence is fragmented and sometimes contradictory. Existing publications typically analyze each of these miRNAs in isolation, in different biological contexts, and without parallel assessment of key Wnt pathway components [10,11,12,13]. Due to discrepancies in previous research findings and a lack of consistent comparative evaluation, we considered miR-106a-5p and miR-375-3p as biologically plausible but insufficiently characterized regulators of Wnt-related signaling in prostate disease. Based on this rationale, both miRNAs were selected for our study alongside β-catenin, Fzd8, β-catenin and cyclin D1, enabling an integrated assessment of transcript and protein expression patterns in BPH and prostate adenocarcinoma specimens.

Therefore, the aim of this study was to compare the expression of miR-106a-5p, miR-375-3p and selected Wnt/β-catenin-related proteins between benign prostatic hyperplasia and prostate adenocarcinoma. Rather than assessing direct biological interactions, our objective was to identify whether these molecules show disease-specific expression patterns that could support their future evaluation as potential discriminators between benign and malignant prostate conditions.

## 2. Results

A total of 60 archival tissue blocks, fixed in formalin and embedded in paraffin, were included in the present study. The sample comprised 30 prostate cancers and 30 cases of BPH. Patients with prostate adenocarcinoma had a mean of 67.3 ± 8.0 years, which did not differ significantly from BPH (69.0 ± 7.5 years).

The clinicopathological characteristics of the studied patients assigned to the BPH and prostate adenocarcinoma groups are presented in Table 1.

**Figure 1:**
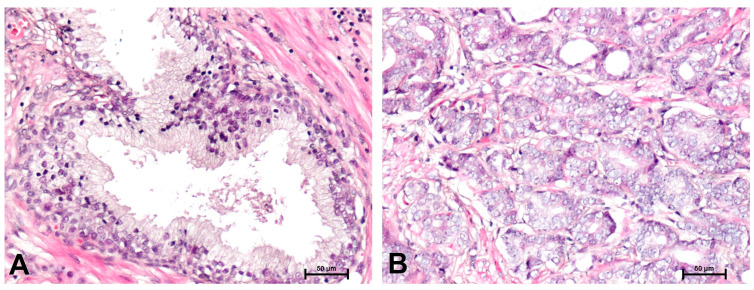
H + E staining in: (**A**) BPH and (**B**) prostate adenocarcinoma. Scale bars: 50 μm.

Histological examination using hematoxylin and eosin staining demonstrated that in BPH samples (Figure 1A), glandular hyperplasia with epithelial proliferation was observed. The glands were characterized by preserved two-layered epithelium and the presence of basal cells. In contrast, a representative prostate cancer sample (Figure 1B) showed features of acinar adenocarcinoma consistent with Gleason pattern 3, defined by loss of the basal cell layer, numerous neoplastic glands with irregular contours, and nuclei with prominent nucleoli.

Analysis of the relationship between PSA levels and the type of histopathological changes revealed no significant differences between patients with BPH and prostate adenocarcinoma (*p* > 0.05). Additionally, in the group of patients with prostate cancer, no significant correlation was found between PSA levels and Gleason grade (*p* > 0.05). The results of these analyses are presented in Table 2.

### 2.1. Immunohistochemical Evaluation

**Figure 2:**
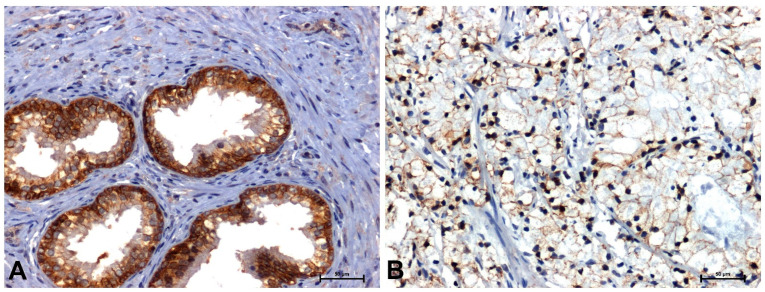
Immunolabeling of β-catenin in: (**A**) BPH and (**B**) prostate adenocarcinoma. Scale bars: 50 μm.

**Figure 3:**
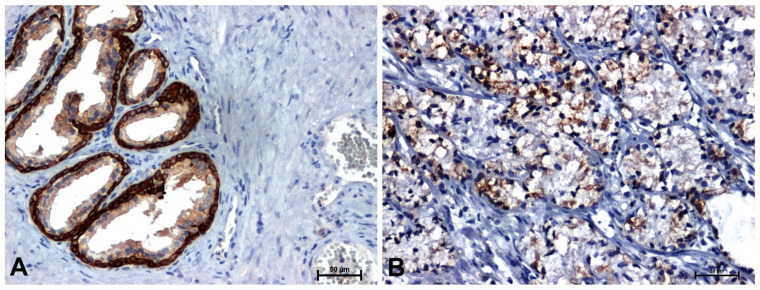
Immunoidentification of Fzd8 in: (**A**) BPH and (**B**) prostate cancer. Scale bars: 50 μm.

**Figure 4:**
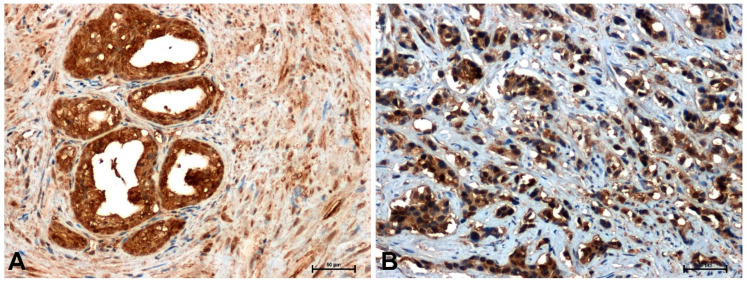
Immunoreactivity of Wnt5a in: (**A**) BPH and (**B**) prostate adenocarcinoma. Scale bars: 50 μm.

**Figure 5:**
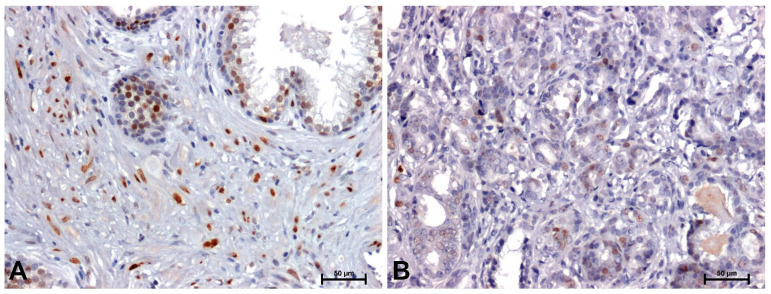
Immunodetection of cyclin D1 in: (**A**) BPH and (**B**) prostate cancer. Scale bars: 50 μm.

The antibodies used in the immunohistochemical reaction (β-catenin, Fzd8, Wnt5a and cyclin D1) gave a positive result in all sections examined (Figure 2, Figure 3, Figure 4 and Figure 5 and Table 1).

Much stronger immunoreactivity was demonstrated in sections of the prostate with benign hyperplasia (Figure 2A, Figure 3A, Figure 4A and Figure 5A), compared to the reaction observed in prostate adenocarcinoma (Figure 2B, Figure 3B, Figure 4B and Figure 5B).

In BPH, a strong reaction was demonstrated showing β-catenin and Fzd8, especially in the membrane of glandular epithelial cells of the prostate (Figure 2A and Figure 3A), while in the tumor sections, a positive reaction was found in the nuclei of some cells (Figure 2B and Figure 3B).

Immunodetection of Wnt5a protein showed strong staining in the cytoplasm of epithelial cells and a much weaker positive reaction in the stroma in BPH sections (Figure 4A). Immunodetection of the Wnt5a receptor showed a positive reaction, with a high intensity in the tumor cells, while in the tumor stroma the result was negative or very weak (Figure 4B).

The weakest immunohistochemical reaction was observed with the anti-cyclin D1 antibody. In BPH glandular cells, a relatively strong positive reaction was observed (Figure 5A), whereas in neoplastic cells the reaction intensity was much weaker (Figure 5B).

Assessment of immunohistochemical staining intensity through digital image analysis, corroborated by statistical testing, confirmed lower immunoreactivity of β-catenin, Fzd8, Wnt5a and cyclin D1 in prostate adenocarcinoma compared to benign prostatic hyperplasia. Data are shown as medians with minimum and maximum values (Table 3).

Statistical analysis showed co-expression between all parameters we examined. Statistically significant results are marked with an asterisk * (*p* ˂ 0.05). As for the relationships between the tested proteins, in the benign prostatic hyperplasia and prostate adenocarcinoma, both positive and negative correlations were found between each protein studied. The results of the correlation analysis between the β-catenin, Fzd8, Wnt5a and cyclin D1, divided into benign prostatic hyperplasia and prostate adenocarcinoma, are presented in Table 4 and Table 5.

### 2.2. Real-Time PCR Analysis

**Figure 6:**
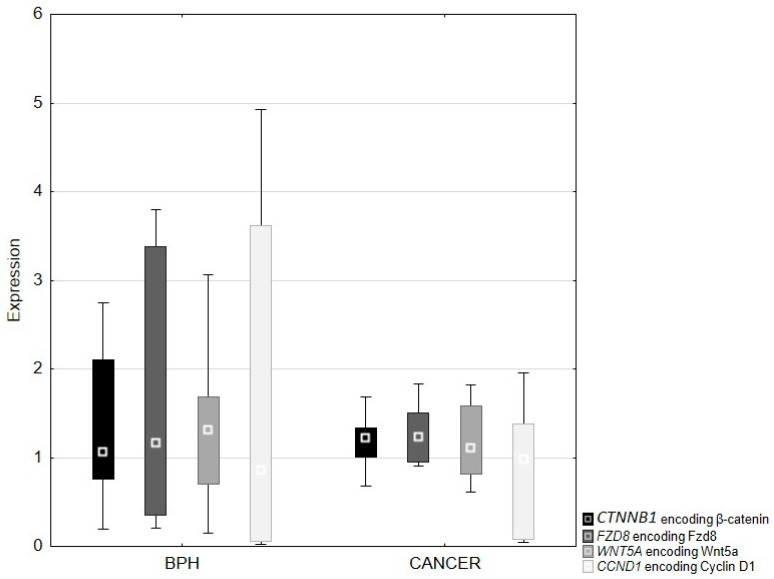
Expression of genes coding β-catenin (*CTNNB1*), Fzd8 (*FZD8*), Wnt5a (*WNT5A*) and cyclin D1 (*CCND1*) in BPH and prostate adenocarcinoma. Box-and-whisker plots show the median, interquartile range, and minimum–maximum values. Statistical comparisons were performed using the Mann–Whitney U test and reflect differences in median expression levels; no statistically significant differences were observed between the groups.

QRT-PCR analysis revealed lower expression of the β-catenin, Fzd8, Wnt5a and cyclin D1 genes in prostate cancer compared to benign prostatic hyperplasia tissues. Differences in the expression of the studied genes between BPH and prostate adenocarcinoma were not statistically significant (Figure 6). Although the expression ranges of the analyzed genes were wide in both groups, the median expression levels did not differ significantly between BPH and prostate adenocarcinoma, as confirmed by the Mann–Whitney U test.

### 2.3. Digital PCR

**Figure 7:**
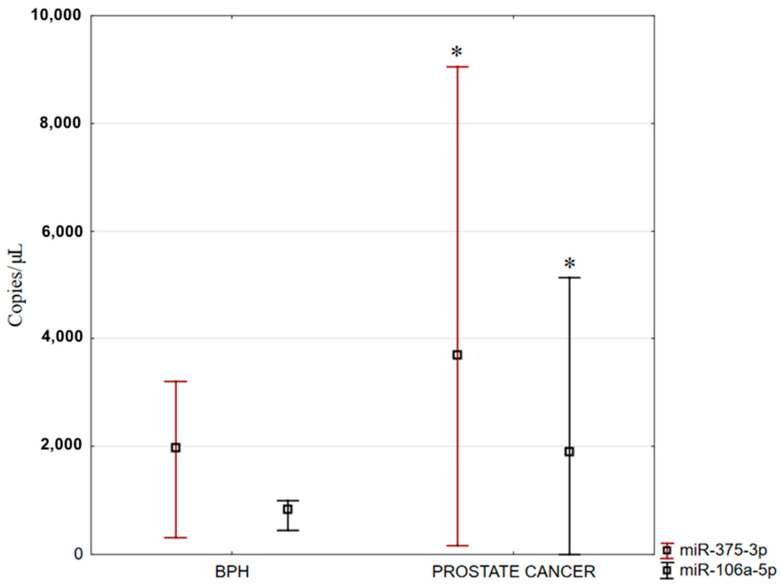
Expression levels of miR-375-3p and miR-106a-5p in benign prostatic hyperplasia and prostate adenocarcinoma. Data are shown as medians with minimum and maximum values; * *p* < 0.05 prostate adenocarcinoma vs. BPH.

A reaction mixture lacking a template—referred to as the no-template control (NTC)—was included as a negative control in the studies. For both analyzed miRNAs, the NTC yielded a fluorescence value of zero. Among the various parameters assessed in miRNA quantification, the most critical is the concentration, expressed as copies/μL. The evaluated miRNAs exhibited differential fluorescence intensities between BPH and prostate adenocarcinoma samples. Specifically, fluorescence signals of both miR-106a-5p and miR-375-3p were significantly higher in prostate adenocarcinoma tissues compared to BPH (Figure 7). The expression levels of these miRNAs in prostate adenocarcinoma were normalized against the values obtained for BPH.

## 3. Discussion

Prostate adenocarcinoma is one of the most common malignant neoplastic diseases in men, which significantly affects the increase in mortality among this group worldwide. A major clinical challenge is to precisely distinguish prostate adenocarcinoma from BPH, given the distinct treatment protocols and management strategies for each condition. Consequently, further gaining insight into the molecular mechanisms responsible for prostate adenocarcinoma and BPH development is of critical importance [14].

Literature reports indicate that certain miRNAs play a key role in the development of prostate adenocarcinoma. So far, no miRNAs with sufficient sensitivity have been implemented in clinical and diagnostic practice that could serve as noninvasive markers for the diagnosis of prostate adenocarcinoma and replace the currently used but imperfect PSA marker [1,8].

Recent studies have shown that certain miRNAs influence cancer cell growth by regulating the Wnt/β-catenin signaling pathway [15,16]. In this context, examining the expression of selected miRNAs along with Wnt-related genes may help to characterize molecular differences between prostate adenocarcinoma and BPH.

The selection of miR-106a-5p and miR-375-3p was based on previous literature demonstrating their dysregulation in prostate cancer and describing their potential involvement in pathways relevant to tumor biology, including the Wnt/β-catenin axis in other cancer types. Therefore, our experimental focus on these two miRNAs was based on existing biological and clinical literature [10,11,12,13].

It has been demonstrated that miR-106a-5p can act as both an oncogene and a tumor suppressor in prostate cancer [17,18,19]. Our study results demonstrated statistically significantly higher miRNA expression in prostate adenocarcinoma compared to BPH.

Similarly, Lu et al. [17] also showed higher expression of miR-106a-5p in prostate cancer compared to non-cancerous tissue, suggesting its oncogenic role in prostate cancer.

A research team led by Shen et al. [19] demonstrated that overexpression of miR-106a in prostate cancer induces apoptosis and inhibits cell proliferation and migration by targeting the 3′-UTR of interleukin-8 mRNA. According to these authors, proliferation and invasive processes in prostate cancer cells are inhibited despite elevated miR-106a levels, suggesting a complex and dual function of miR-106a-5p in the pathogenesis of prostate adenocarcinoma.

Another study of plasma from patients with prostate adenocarcinoma demonstrated reduced expression of miR-106a-5p compared with men with benign prostatic hyperplasia [8]. These differences may be due to the different regulation of miR-106a-5p between the tissue environment and body fluids such as plasma, which has important implications for the potential use of this miRNA as a marker and emphasizes the cautious interpretation of study results.

Our study also showed significantly higher expression of miR-375 in prostate adenocarcinoma compared to non-cancerous tissues.

MiR-375 has been identified as an important tumor suppressor that has been shown to be dysregulated in various types of cancer [20,21].

Studies have shown reduced expression of miR-375 and a suppressor role in many different types of cancers, while higher levels of miR-375-3p were found in prostate cancer compared to healthy tissues [22].

Also, the studies conducted by Abramovic et al. [23] showed higher expression of miR-375-3p in plasma of prostate cancer patients compared to patients with benign hyperplasia. According to the authors of these studies, miR-375-3p measured in blood is a better biomarker for differentiating prostate cancer from benign hyperplasia than PSA.

Activation of the canonical Wnt/β-catenin pathway in prostate cancer is observed primarily in the late phase of this disease and causes resistance to therapy [24].

Our results showed lower expression of Wnt pathway-related proteins—β-catenin, Fzd8, Wnt5a and cyclin D1—in prostate cancer compared to BPH tissues, but the differences were not statistically significant.

Accumulation and nuclear translocation of β-catenin lead to activation of the Wnt pathway and changes in the expression of genes responsible for cell proliferation [25].

Our results showed lower β-catenin expression in prostate adenocarcinoma compared with benign prostatic hyperplasia. In contrast, Jung et al. [26] demonstrated higher β-catenin expression in patients with prostate cancer. Discrepancies in results may be due to differences in the stage of cancer advancement or molecular differentiation of prostate cancer.

Importantly, in the cancer tissues that we studied, β-catenin was located exclusively in the cell nucleus, which may suggest the activation of the canonical Wnt pathway, despite the lower level of β-catenin expression. This may indicate that the subcellular localization of this protein, and not only its level, is an important factor responsible for the functional activity of the Wnt pathway. It is likely that in the studied cases, there is transcriptional activation of β-catenin, accompanied by a simultaneous reduction in its level in the cytoplasm, which could have contributed to the weakening of the immunohistochemical reaction result.

Fzd8 receptors induce cell signal transduction by binding Wnt ligands. In a recent study on long non-coding RNAs (lncRNAs), LINC00115 was shown to induce prostate cancer cell proliferation via the miR-212-5p/FZD5/Wnt/β-catenin axis, in which Fzd8 leads to the activation of the canonical Wnt pathway [27]. This study and our own results support the hypothesis that the Wnt pathway and its components are crucial in the development of prostate adenocarcinoma.

The Wnt5a protein is a ligand for the Fzd8 receptor, activating the classical Wnt signaling pathway. It turns out that this protein plays a dual role in prostate cancer. It can promote cancer progression through epithelial–mesenchymal transition or interactions with the androgen receptor pathway, or act as a suppressor under certain conditions by inhibiting the Wnt/β-catenin pathway [28].

Studies conducted by a team led by Xie [28] have shown that the function of the Wnt5a protein in prostate cancer depends on the stage of the disease and the tissue context. Due to the growing number of reports indicating the significant role of individual Wnt pathway components in cancer pathogenesis, the assessment of Wnt5a expression in prostate adenocarcinoma and BPH may be of great importance in guiding new therapies directly targeting the regulation of Wnt pathway activity.

In our study, we showed reduced expression of Wnt5a in prostate adenocarcinoma, which may indicate a suppressor role of the protein in this tumor. Thiele et al. [29] confirmed that in prostate cancer, Wnt5a acting through FZD5 receptors leads to reduced tumor cell proliferation and even induces cancer cell apoptosis.

Cyclin D1 is a proto-oncogene protein that is currently the subject of intensive research due to its key role in the cell cycle and the fact that its disruption can lead to cancer development. Our study demonstrated slightly lower cyclin D1 expression in prostate cancer compared to BPH.

The few studies on cyclin D1 expression in prostate adenocarcinomas have presented conflicting results [30,31]. Wang et al. [30] observed overexpression of cyclin D1 in prostate adenocarcinoma, while the research team led by Yin [31] reported reduced expression of this protein in prostate cancer, highlighting the heterogeneous nature of cyclin D1 regulation in prostate cancer progression.

In benign prostatic hyperplasia, we found a predominance of negative correlations between the expression levels of the studied proteins. This means that when the level of one protein increased, the level of another tended to decrease. Such correlations may suggest the presence of regulatory mechanisms, in which some proteins inhibit or neutralize the activity or expression of others. This pattern may reflect a biological balance typical of non-malignant tissue, in which various signaling pathways may function to maintain tissue homeostasis and prevent excessive cellular proliferation.

In prostate adenocarcinoma, correlation analysis revealed a predominance of positive associations between the studied proteins. This indicates that increased expression of one protein is accompanied by a similar increase in the expression of another. Such coordinated expression patterns may reflect the activation of interconnected biological pathways, in which proteins act synergistically to promote tumor-related processes. Differences in the correlations between studied proteins in BPH and prostate adenocarcinoma may suggest different pathological mechanisms underlying these conditions. Furthermore, they may indicate different protein interaction profiles in benign hyperplasia and prostate cancer.

MiRNAs can influence the activity of the Wnt/β-catenin pathway by altering the expression of Wnt ligands, β-catenin protein, receptors, or β-catenin-interacting complexes [32]. The research team led by Shen [19] did not prove that miR-106a-5p directly affects the Wnt/β-catenin pathway in prostate cancer, but they did demonstrate a role for this miRNA in association with interleukin-8, which may affect the activity of the canonical Wnt pathway. In addition, miR-106a-5p levels are regulated by c-Src/PI3K/Akt 30, which may interact with the Wnt pathway.

A more direct effect on the Wnt/β-catenin pathway has been demonstrated for miR-375-3p. In a study of gastric cancer, Guo et al. [33] showed that miR-375-3p inhibits the Wnt pathway by reducing the expression of the YWHAZ protein, which regulates proliferation and apoptosis. This is valuable information on how miR-375-3p affects the activity of the Wnt pathway, influencing the mechanisms occurring in cancer cells. The canonical pathway is involved in cell adhesion processes, which are crucial in the context of metastasis and tumor invasion. It is possible that the loss of the adhesion protein E-cadherin promotes β-catenin release and leads to the activation of the Wnt pathway [32,34]. The present information confirms the influence of miR-375 on the activity of the Wnt/β-catenin pathway; however, this is a complex regulation full of interactions between the involved components, which requires further analysis.

Our study results, indicating lower expression of β-catenin, Fzd8, Wnt5a and cyclin D1, may suggest attenuated activity of the Wnt/β-catenin pathway in prostate adenocarcinoma compared to benign prostatic hyperplasia. This is important in light of literature reports indicating a key role for activation of the canonical pathway in the progression of prostate adenocarcinoma and its bone metastases [35,36].

In this study, qRT-PCR analysis did not reveal differences in the expression of the studied genes between BPH and prostate adenocarcinoma, whereas IHC results showed differential expression at protein level. Data from the scientific literature indicate that many genes exhibit discordant mRNA–protein relationships in prostate tissue, which may be due to post-transcriptional regulation, differences in protein stability, and subcellular localization. Furthermore, it is possible that the elevated fluorescence levels of miR-106a-5p and miR-375-3p in prostate cancer may inhibit translation or promote degradation of target mRNAs involved in the Wnt/β-catenin axis. Immunohistochemistry provides spatial information, for example, regarding epithelial versus stromal compartments and subcellular localization within the prostate, whereas qRT-PCR measures averaged mRNA levels across all cell types present in tissue homogenates. These considerations indicate that the combined use of immunohistochemistry and qRT-PCR provides complementary rather than overlapping information and helps explain why statistically significant differences between BPH and prostate adenocarcinoma were detected at the protein level but not at the mRNA level [13,37,38].

In our study, we also analyzed the relationship between serum PSA values, histopathological diagnosis, and Gleason grade, and found no statistically significant differences or correlations. Our observations are consistent with previous reports indicating the limited ability of PSA to clearly distinguish BPH from prostate cancer and the lack of a strong, consistent correlation between PSA levels and Gleason score in some cohorts [37,39]. At the same time, other studies have reported moderate or significant associations between PSA levels and tumor grade in larger or differently selected populations, suggesting that the heterogeneity of findings may result from differences in sample size, inclusion criteria, the use of PSA density, or measurement methodology [40]. Therefore, given the pilot character of our cohort, we emphasize the need for further validation studies in larger and well-characterized populations, as well as the potential inclusion of MRI-based metrics in future analyses.

The relationships between the analyzed miRNAs and the Wnt/β-catenin pathway, as reported in the literature, are supported by heterogeneous and sometimes conflicting evidence. Our study did not investigate functional interactions, but the parallel expression changes observed may help identify molecular patterns characteristic of prostate adenocarcinoma. Further detailed studies, including functional assays, are necessary to clarify whether differentially expressed miRNAs affect Wnt-related targets in prostate cancer. In this context, particular attention should be paid to the study of β-catenin, which exhibits dual function, and context-dependent protein Wnt5a in various tumor microenvironments.

We are aware of the limitations of our study that should be taken into account. Due to the relatively small sample size, these data should be considered preliminary and worth validating on larger data sets. Our finding that adenocarcinoma tissues exhibit significantly higher expression of miR-106a-5p and miR-375-3p and lower expression of canonical Wnt signaling pathway components is potentially interesting but requires cautious interpretation. In further studies, we intend to validate it in larger cohorts of patients with prostate cancer and BPH.

## 4. Materials and Methods

### 4.1. Sample Collection

The research was carried out using postoperative specimens obtained from 30 individuals diagnosed with prostatic adenocarcinoma and 30 patients with benign prostatic hyperplasia, all treated at the Urology Department of the Medical University of Bialystok. Ethical clearance for the study was granted by the Bioethics Committee of the Medical University of Bialystok (APK.002.22.2024; approval date: 18 January 2024), and written informed consent was obtained from each person to participate in the study. Patients were assigned to the appropriate experimental groups based on variables such as age, serum PSA level and histopathological result with Gleason score. The research material consisted of fragments of prostatic adenocarcinoma and benign prostatic hyperplasia collected during adenomectomy or transurethral resection of the prostate.

Each sample collected for testing from patients was evaluated and classified by a pathologist as part of routine diagnostic procedures, in accordance with current WHO criteria. The diagnosis of BPH and prostate adenocarcinoma was made based on classical histopathological evaluation of hematoxylin and eosin-stained sections, supplemented with immunohistochemistry in diagnostically equivocal cases. For the purposes of this study, BPH samples were included only when morphology showed typical glandular–stromal hyperplasia and an intact, continuous basal cell layer confirmed by IHC. Adenocarcinoma samples were obtained from separate cases, demonstrating loss of the basal layer, cytological atypia, and infiltrative growth, and the histological grade was determined according to the Gleason system. Because basal cell loss and a positive AMACR test result are not always absolutely specific markers, diagnostically equivocal cases were reanalyzed and excluded from the final cohort.

Tissue samples were promptly preserved either in 10% buffered formalin followed by routine paraffin embedding or in RNA-later solution (AM7024, Thermo Fisher Scientific, Waltham, MA, USA) and subsequently stored at −80 °C. Paraffin-embedded blocks were sectioned at a thickness of 4 μm, stained with hematoxylin and eosin for general histopathological assessment, and further processed for immunohistochemical detection of β-catenin, Fzd8, Wnt5a, and cyclin D1. Materials stored in RNA-later were analyzed by real-time PCR to determine the transcriptional levels of the genes encoding β-catenin, Fzd8, Wnt5a, and cyclin D1.

### 4.2. Immunohistochemistry

Immunohistochemical analysis was carried out using the EnVision technique, following the procedure previously outlined by Kasacka et al. [41]. The immunohistochemistry assays were performed with the REAL™ EnVision™ Detection System, Peroxidase/DAB, Rabbit/Mouse (K5007; Dako Cytomation, Glostrup, Denmark). Immunostaining was conducted according to the following protocol: paraffin-embedded sections were deparaffinized and hydrated in pure alcohols.

For antigen retrieval, the sections were subjected to pretreatment in a pressure chamber heated for 1 min at 21 psi (one pound force per square inch (1 psi) equates to 6.895 kPa; the conversion factor was provided by the NPL (United Kingdom National Physical Laboratory)) at 125 °C with the use of Target Retrieval Solution Citrate, pH 6.0 (S2369; Dako Cytomation, Glostrup, Denmark) for β-catenin, Fzd8, Wnt5a, and cyclin D1. Following cooling to ambient temperature, the tissue sections were treated with a Peroxidase Blocking Reagent (S 2023 Agilent Technologies Denmark, Glostrup, Denmark) for 10 min to block endogenous peroxidase activity. Subsequently, sections were incubated with primary antibody for β-catenin (Mouse monoclonal to β-catenin, ab32572, ABCAM Discovery Drive, Cambridge, UK), Fzd8 (Rabbit monoclonal to Fzd8, ab155093, ABCAM Discovery Drive, Cambridge, UK), Wnt5a (Rabbit polyclonal to Wnt5a, ab235966, ABCAM Discovery Drive, Cambridge, UK) and cyclin D1 (Mouse monoclonal to cyclin D1, ab273608, ABCAM, Cambridge, UK). All antibodies were previously diluted in EnVision FLEX Antibody Diluent (K8006 Agilent Technologies, Santa Clara, CA, USA) at a ratio of 1:2000 for β-catenin, 1:400 for Fzd8 antibody, 1:100 for Wnt5a and 1:100 for cyclin D1. Tissue sections were incubated overnight at 4 °C in a humidified chamber, followed by exposure to a secondary antibody conjugated with a horseradish peroxidase–labeled polymer. Antibody binding was visualized using a 1 min reaction with liquid 3,3′-diaminobenzidine substrate chromogen. Subsequently, the sections were counterstained with hematoxylin QS (H-3404, Vector Laboratories; Burlingame, CA, USA), mounted, and examined under a light microscope. Between each step, appropriate rinsing with Wash Buffer (S3006; Dako Cytomation Glostrup, Denmark) was carried out. To verify specificity and exclude non-specific antibody interactions with the examined tissue, negative controls were included. Negative control reactions were performed in which the specific antibody of the detected antigens (β-catenin, Fzd8, Wnt5a and cyclin D1) was replaced with normal rabbit serum (Vector Laboratories, Newark, CA, USA) with the proper dilution. No positive immunoreactivity was observed. Staining outcomes were evaluated with an Olympus BX43 light microscope (Olympus Corp., Tokyo, Japan) equipped with an Olympus DP12 digital camera and subsequently documented.

### 4.3. Quantitative Analysis

For each patient, twelve sections of benign prostatic hyperplasia and carcinoma tissue were analyzed, with three sections allocated to the immunostaining of β-catenin, Fzd8, Wnt5a, and cyclin D1, respectively.

In BPH, only the luminal cells of the glandular epithelium were analyzed, excluding basal and stromal cells. Similar criteria were applied to prostate adenocarcinoma, where tumor epithelial cells were evaluated.

From every section, five randomly selected microscopic fields (0.785 mm^2^ each, captured at 200× magnification using a 20× objective and 10× eyepiece) were documented with an Olympus DP12 camera. Digital images were subsequently subjected to morphometric assessment with NIS Elements AR 3.10 (Nikon Instruments, Tokyo, Japan) image analysis software (version 3.10). In each field, the intensity of immunohistochemical labeling for all antibodies was quantified on a grayscale ranging from 0 to 256, where 0 corresponded to completely white/light pixels, and 256 represented completely black pixels.

### 4.4. Real-Time PCR

Samples of benign prostatic hyperplasia and cancer were obtained from every individual and inserted in an RNA-later solution.

Total RNA was isolated with the NucleoSpin^®^ RNA Isolation Kit (Machery-Nagel, Düren, Germany). The concentration and purity of the RNA were assessed using a NanoDrop 2000 spectrophotometer (Thermo Fisher Scientific, Waltham, MA, USA). Only samples with A260/280 ratios between 1.8 and 2.0 were included in further analyses. The concentration of RNA was then standardized before reverse transcription: for mRNA studies, 1 µg of total RNA from each sample was used for cDNA synthesis; for miRNA studies, the RNA concentration was adjusted to 5 ng/µL, and 2 µL of RNA was used per reaction.

Subsequently, 1 μg of total RNA was reverse-transcribed into cDNA with the iScript™ Advanced cDNA Synthesis Kit for RT-qPCR (BIO-RAD Laboratories, Hercules, CA, USA). The cDNA synthesis was carried out in a final reaction volume of 20 μL employing a SureCycler 8800 thermal cycler (Agilent Technologies, Santa Clara, CA, USA). For reverse transcription, the mixtures were incubated at 46 °C for 20 min, then heated to 95 °C for 1 min, and finally cooled quickly at 4 °C. Quantitative real-time PCR reactions were performed using Stratagene Mx3005P (Agilent Technologies, Santa Clara, CA, USA) with the SsoAdvanced™ Universal SYBER^®^ Green Supermix (BIO-RAD Laboratories, Hercules, CA, USA). Specific primers for β-catenin (*CTNNB1*), Fzd8 (*FZD8*), Wnt5a (*WNT5A*), cyclin D1 (CCND1) and GAPDH (*GAPDH*) were created by BIORAD Company.

In the present study, *GAPDH* was used as a reference gene because it is widely applied and considered suitable for normalizing gene expression analyses in both BPH and prostate adenocarcinoma. To quantify transcript abundance, standard curves were generated individually for each gene using serial dilutions of PCR products. These PCR products were obtained by amplifying cDNA with the following gene-specific primers: *CTNNB1* (qHsaCED0046518, BIO-RAD, Laboratories, Hercules, CA, USA), *FZD8* (qHsaCED0019650, BIO-RAD Laboratories, Hercules, CA, USA), *WNT5A* (qHsaCID0012240, BIO-RAD Laboratories, Hercules, CA, USA), *CCND1* (qHsaCID0013833, BIO-RAD Laboratories, Hercules, CA, USA) and *GAPDH* (qHsaCED0038674, BIO-RAD Laboratories, Hercules, CA, USA). Quantitative real-time PCR (qRT-PCR) was performed in duplicate in a final reaction volume of 10 μL under the following cycling conditions: initial polymerase activation for 2 min at 95 °C, followed by 40 cycles of denaturation for 5 s at 95 °C and annealing/extension for 30 s at 60 °C. Control reactions included no-RT controls, template-free reactions, and melting curve analysis to verify amplification specificity and confirm the production of a single PCR product.

### 4.5. miRNA Selection

The selection of miRNAs for experimental validation was based on a review of the literature suggesting an association with prostate cancer and/or components of the Wnt/β-catenin signaling pathway. Based on this review, miR-106a-5p and miR-375-3p were considered as priorities for experimental analysis in our considerations.

### 4.6. Extraction of miRNA from Tissues

miRNA was extracted from benign prostatic hyperplasia and prostate adenocarcinoma tissue samples using the miRNeasy Tissue/Cells Advanced Micro Kit (Qiagen, Copenhagen, Denmark, cat. no. 217684) according to the protocol prepared by the manufacturer. A section of 4 mm^3^ was removed from each tissue specimen and put into a 1.5 mL reaction tube along with 60 μL of lysis solution that contained 1% β-mercaptoethanol. Tissue samples were homogenized using a disposable polypropylene pestle and subsequently suspended in 700 μL of QIAzol Lysis Reagent (Qiagen, Copenhagen, Denmark, cat. no. 79306). All subsequent steps were performed following the manufacturer’s protocol. The RNA was eluted in 40 μL of RNase-free water (Qiagen, Copenhagen, Denmark, cat. no. 129112).

### 4.7. Quantification of RNA

The purity, concentration, and potential contaminants of the extracted miRNA were assessed using a NanoDrop spectrophotometer (Thermo Fisher Scientific, Waltham, MA, USA).

### 4.8. cDNA Synthesis

Reverse transcription of RNA was performed using the miRCURY LNA RT Kit (Qiagen, cat. no. 339340). For fresh-frozen benign prostatic hyperplasia and prostate adenocarcinoma tissues, RNA concentrations were standardized to 5 ng/μL, and 2 μL of each sample was incorporated into a total reaction volume of 10 μL, which also contained 0.5 μL of UniSp6 RNA spike-in.

### 4.9. Digital PCR (dPCR) Procedure

Digital PCR reactions were conducted using the miRCURY LNA miRNA PCR Assays kit (Qiagen, Hilden, Germany, cat. no. 39306) in a 96-well plate format (Qiagen, Hilden, Germany, cat. no. 250021) and the QIAcuity One nucleic acid detection instrument using the QIAcuity Software Suite 2.1.8.20 (Qiagen, Hilden, Germany). The reaction mixture was prepared following the manufacturer’s instructions, comprising 4 μL of 3× EvaGreen PCR Master Mix (Qiagen, Hilden, Germany, cat. no. 250111), 1.2 μL of 10× miRCURY LNA PCR Assay (Qiagen, Hilden, Germany, cat. no. 339306), 3 μL of cDNA template (Qiagen, Hilden, Germany and 3.8 uL of RNase-free water (Qiagen, Hilden, Germany, cat. no. 129112). Total reaction volume was 12 uL. 96-well nanoplates with 8.500 partitions were used for the studies. The miRNAs analyzed in this study were 106a-5p (Qiagen, Hilden, Germany, cat. no. YP00204563) and 375-3p (Qiagen, Hilden, Germany, cat. no. YP00204362). The prepared reaction mix was initially dispensed into a standard PCR plate, from which the contents of each well were transferred to a QIAcuity Nanoplate (Qiagen, Hilden, Germany, cat. no. 250021). Digital PCR was carried out under cycling conditions recommended by the manufacturer: an initial heat activation at 95 °C for 2 min, followed by 40 cycles of 15 s denaturation at 95 °C and 1 min annealing/extension at 60 °C, and a final cooling step at 40 °C for 5 min. Upon completion, raw data were exported to the QIAcuity Software Suite 2.1.8.20 (Qiagen, Hilden, Germany) for analysis.

### 4.10. Statistical Analysis

All collected data were subjected to statistical analysis using the Statistica software package, Version 14.1. Statistical analysis revealed a lack of normality in the distribution of the obtained results. Results regarding immunoreactivity and expression of the analyzed parameters are expressed as medians accompanied by their respective minimum and maximum values. As distribution of the obtained data deviated from normality, the non-parametric Mann–Whitney U test was used to assess statistical significance of differences between benign prostatic hyperplasia and prostate adenocarcinoma. Differences were considered statistically significant when the *p*-value was less than 0.05 (*p* < 0.05).

Patient age data were expressed as means, and PSA values as medians. Differences between groups were assessed using the Kruskal–Wallis test, with *p* < 0.05 considered statistically significant. Correlations between PSA levels in BPH and prostate adenocarcinoma, as well as between PSA values and Gleason score in prostate cancer, were evaluated using Spearman rank correlation test (R—correlation coefficient).

Regarding the type of correlation used, linear regression analysis was applied to compare the relationships between the β-catenin, Fzd8, Wnt5a and cyclin D1 in BPH and cancer tissue. The main goal of the analysis was to assess the strength and direction of protein correlations. This allowed for detailed insight into how the expression of one protein influences expression of another in benign prostatic hyperplasia and prostate adenocarcinoma. In order to analyze the correlation between the tested proteins, statistical regression analysis was performed. The outcomes of this analysis are presented as the β coefficient, indicating the percentage change in the dependent variable per unit change in the independent variable; r^2^, representing the proportion of variability in one variable explained by the other; and the associated *p*-value for statistical significance. A relationship between two variables was considered statistically significant when the *p*-value corresponding to the β coefficient was less than 0.05.

In the qRT-PCR, relative gene expression levels were determined using the ∆∆Ct method by comparing cycle threshold (Ct) values. All gene expression data were normalized to the housekeeping gene *GAPDH*. For miRNA analysis, expression levels in prostate adenocarcinoma samples were normalized relative to those in benign prostatic hyperplasia tissues.

## 5. Conclusions

In summary, the results of this study showed significantly higher expression of miR-106a-5p and miR-375-3p in prostate adenocarcinoma tissues compared with BPH, accompanied by lower expression of β-catenin, Fzd8, Wnt5a, and cyclin D1. These findings suggest that concurrent alterations in the analyzed miRNAs and Wnt/β-catenin-related genes may reflect underlying molecular differences between prostate adenocarcinoma and BPH. As this was a pilot study, the observed expression patterns should be interpreted as descriptive and correlative rather than mechanistic. Nevertheless, the results obtained in the conducted studies justify further research on the potential biological and diagnostic significance of the tested molecules in differentiating benign and malignant prostate diseases.

Furthermore, no significant differences in serum PSA levels were found between BPH and prostate adenocarcinoma, and no correlations were observed between PSA levels and Gleason grade within the prostate cancer group, confirming the limited specificity of PSA and its inconsistent relationship with tumor aggressiveness. These observations highlight the need for further validation in larger, well-characterized patient cohorts and indicate the potential value of integrating the investigated molecular biomarkers with imaging-based approaches to enhance diagnostic precision in prostate diseases.

## Figures and Tables

**Table 1:** Clinicopathological characteristics of the studied groups.

Characteristics	BPH *n* = 30	Prostate Adenocarcinoma *n* = 30	*p*-Value
Age–average range	69.0 (58–82)	67.3 (52–85)	*p* = 0.782
Gleason score	-		-
Gleason score 6		14 (46.7%)	
Gleason score 7		5 (16.7%)	
Gleason score 8		3 (10%)	
Gleason score 9		8 (26.7%)	
PSA (ng/mL)– medians	6.5 (10.4–2.1)	8.8 (74.4–4.7)	*p* = 0.59

Age presented as mean values; PSA presented as medians (Kruskal–Wallis test).

**Table 2:** Correlations between PSA levels in BPH and prostate adenocarcinoma, and PSA and Gleason score in prostate cancer.

	BPH *PSA* (ng/mL)	Prostate Cancer *Gleason Score*
Prostate cancer PSA (ng/mL)	*p* = 0.645 R = 0.214	*p* = 0.434 R = 0.355

R—rank correlation coefficient (Spearman test).

**Table 3:** The intensity of immunoreaction determining β-catenin, Fzd8, Wnt5a and cyclin D1 in BPH and prostate adenocarcinoma.

Intensity of Immunohistochemical Reaction in BPH and Prostate Adenocarcinoma Scale from 0 (White Pixel) to 256 (Black Pixel)				
	β-Catenin	Fzd8	Wnt5a	Cyclin D1
BPH—luminal cells	134.4 (82.88–149.3)	163.9 (149.0–185.8)	191.0 (182.8–198.8)	74.77 (58.17–98.52)
Prostate cancer	43.21 (18.01–62.44) *	81.63 (59.91–98.93) *	109.6 (94.77–140.3) *	55.45 (38.35–70.59) *

Data are shown as medians with minimum and maximum values; * *p* < 0.05 prostate adenocarcinoma vs. BPH.

**Table 4:** Immunohistochemistry-based correlation analysis of β-catenin, Fzd8, Wnt5a and cyclin D1 in BPH.

BPH				
β-Catenin	Fzd8	Wnt5a	Cyclin D1	
**-**	β = −0.9176 *p* = 0.1264 r^2^ = 0.2668	β = +0.0104 *p* = 0.8780 r^2^ = 0.0031	β = −0.4344 *p* = 0.0188 * r^2^ = 0.5189	β-catenin
	**-**	β = +0.1033 *p* = 0.3791 r^2^ = 0.0978	β = −0.5369 *p* = 0.1400 r^2^ = 0.2512	Fzd8
		**-**	β = −0.8082 *p* = 0.4875 r^2^ = 0.0621	Wnt5a
			**-**	Cyclin D1

Data shown as β coefficient, r^2^ and statistical significance, where * *p* < 0.05.

**Table 5:** Correlation analysis of immunohistochemical expression of β-catenin, Fzd8, Wnt5a and cyclin D1 in prostate adenocarcinoma.

Prostate Adenocarcinoma				
β-Catenin	Fzd8	Wnt5a	Cyclin D1	
**-**	β = +0.0179 *p* = 0.00000 * r^2^ = 0.7236	β = +0.0249 *p* = 0.00000 * r^2^ = 0.8781	β = +0.0555 *p* = 0.0266 * r^2^ = 0.2447	β-catenin
	**-**	β = +0.0209 *p* = 0.00003 * r^2^ = 0.6264	β = −0.0482 *p* = 0.00002 * r^2^ = 0.6550	Fzd8
		**-**	β = −0.0154 *p* = 0.00000 * r^2^ = 0.8781	Wnt5a
			**-**	Cyclin D1

Data shown as β coefficient, r^2^ and statistical significance, where * *p* < 0.05.

## Data Availability

The datasets presented in this article are not readily available because due to technical/time limitations. Requests to access the datasets should be directed to corresponding author.

## Abbreviations

BPH	benign prostatic hyperplasia
dPCR	digital PCR
miRNAs	microRNAs
PSA	prostate-specific antigen
